# Regulation of MUTYH, a DNA Repair Enzyme, in Renal Proximal Tubular Epithelial Cells

**DOI:** 10.1155/2015/682861

**Published:** 2015-10-20

**Authors:** Jianping Lu, Xinxiu Li, Mingcao Zhang, Zhaohong Chen, Yaping Wang, Caihong Zeng, Zhihong Liu, Huimei Chen

**Affiliations:** ^1^Department of Medical Nephrology, Jinling Hospital, Nanjing Clinical School of Southern Medical University, Nanjing 210002, China; ^2^National Clinical Research Center of Kidney Disease, Jinling Hospital, Nanjing University School of Medicine, Nanjing 210002, China; ^3^Jiangsu Key Laboratory of Molecular Medicine, School of Medicine, Nanjing University, Nanjing 210093, China

## Abstract

MUTYH is a DNA repair enzyme that initiates a base excision repair (BER) by recognizing and removing 8-Oxoguanine (8-oxoG) and its paired adenine. We demonstrated that both TGF-*β*1 and H_2_O_2_ treatment led to an increased 8-oxoG in cultured human proximal tubule epithelial (HK-2) cells, while the former induced epithelial-mesenchymal transition and the latter caused cell apoptosis. Without stimulation, HK-2 cells showed MUTYH expression in mitochondria. TGF-*β*1 triggered a transient upregulation of mitochondrial MUTYH and induced the expression of nuclear isoforms, while H_2_O_2 _showed no role on MUTYH expression. Ureteral obstruction (UUO) mice exhibited high 8-oxoG reactivity with tubulointerstitial lesions. After obstruction, the MUTYH expression was increased only in tubules at day 3 and decreased with obvious tubular atrophy at day 10. Particularly, MUTYH was primarily located in normal tubular cytoplasm with a dominant mitochondrial form. A few cells with nuclear MUTYH expression were observed in the fibrotic interstitium. We confirmed that increased MUTYH expression was upregulated and positively correlated with the severity of kidney fibrosis. Thus, renal fibrosis caused a cell-type-specific and time-dependent response of oxidative DNA repairs, even within the same tissues. It suggests that intervention of MUTYH might be effective for therapies.

## 1. Introduction

Renal fibrosis occurs in many forms of chronic kidney disease progressing to end-stage renal disorders (ESRD) [1]. Oxidative stress in kidneys is often suggested to contribute to interstitial fibrosis [2]. DNA is one of the most important biological targets of oxidative stress, and oxidative DNA lesions are a major type of endogenous damage leading to human disorders. The most stable product of oxidative DNA damage is 8-Oxoguanine (8-oxoG) [3, 4], and 8-oxoG has been observed in kidneys in several conditions.

Adenine can pair with 8-oxoG in double-stranded DNA, leading to mispairing during genome replication [5]. If the mismatch is not repaired, the G: C to T: A mutation will be inherited in future cell cycles. Mammalian cells are equipped with elaborate means to minimize 8-oxoG accumulation in DNA [6, 7]. The human MutY homolog (*MUTYH*) initiates a base excision repair (BER) by recognizing and removing 8-oxoG and its paired adenine. Mutated BER proteins are reported to increase the risk of accumulating 8-oxoG in mitochondrial DNA (mtDNA) as well as nuclear DNA (nDNA). Mutant mice lacking MUTYH exhibit increased spontaneous mutation rates and susceptibility to carcinogenesis, with increased accumulation of 8-oxoG in DNA.

Oxidative stress is important in the etiology of several renal disorders, and 8-oxoG levels are increased in the kidneys of patients with several diseases, including diabetic nephropathy (DN) [8]. We therefore explored the possible association between MUTYH and ESRD and showed an increased risk of germline MUTYH polymorphisms during ESRD development [9]. This implied that MUTYH might be implicated in the pathogenesis of renal fibrosis. However, how this enzyme influences fibrosis is poorly understood. In addition, there are more than ten splice variants in mammalian cells [10], which localize to either nuclei or mitochondria. The 52 or 53 kDa MUTYH variant is generally localized in nuclei, whereas the 57 kDa MUTYH form localizes in mitochondria. The two forms are involved in distinct signaling pathways. The exact MUTYH form involved in renal fibrosis is not yet known.

Unilateral ureteral obstruction (UUO) is a widely used* in vivo* model of renal fibrosis [11]. It is a good example of the role of epithelial-mesenchymal transition (EMT) and renal oxidative stress in chronic progressive renal disease. The tubular epithelial cells play a key role in this process, and the immortalized HK-2 cell line is often used to study the mechanism of renal fibrosis* in vitro* [12]. Transforming growth factor-*β*1 (TGF-*β*1) induced EMT and hydrogen peroxide induced renal tubular cell apoptosis were used to study the association with MUTYH. The* in vivo* and* in vitro* responses of MUTYH (mitochondrial and/or nuclear forms) in renal fibrosis were investigated in the present study.

## 2. Materials and Methods

### 2.1. Reagents

The primary antibodies used in this study were mouse monoclonal anti-8-oxoG (JaICA, Shizuoka, Japan); rabbit anti-MUTYH (BS2535, Bioworld Technology); mouse monoclonal anti-alpha smooth muscle actin (ab119952, Abcam); and anti-GAPDH (AP0066, Bioworld Technology). Recombinant human transforming growth factor beta-1 (TGF-*β*1) was purchased from R&D (240-B-010, R&D Systems).

### 2.2. Patients and Materials

Needle biopsies from renal tissues were analyzed in the present study. The patients with diabetic nephrology (DN) were randomly chosen from the Biobank of the National Clinical Research Center of Kidney Diseases, Jinling Hospital. The biopsy specimens were processed according to standard procedures and divided into two groups according to the tubular atrophy (IFTA) scores [13]. The mild renal fibrosis group (*n* = 5) was defined as Grades 0~1 and the severe group (*n* = 5) as Grades 2~3. The study protocol was approved by the Ethics Committee of Jinling Hospital, Nanjing University School of Medicine, Nanjing, China.

For the UUO models, C57BL/6 mice (8 weeks old, male) were purchased from the Experimental Laboratory of Animal Models (Nanjing, China). The test group was anesthetized and the left ureter was ligated (UUO group, *n* = 8), while controls were anesthetized and manipulated without ligation (sham group, *n* = 8). The study was approved by the Institutional Animal Care and Use Committee of Nanjing University School of Medicine.

Injured tubular cells were further investigated using two* in vitro* models. Immortalized proximal tubular epithelial cells from the normal adult human kidney (HK-2) were cultured in DMEM/F12 medium (1 : 1, Gibco) supplemented with 10% FBS. In one model, HK-2 cells were treated with 5 ng/mL of recombinant TGF-*β*1 for 24 h, 48 h, and 72 h, as previously described [14]. In the other model, HK-2 cells were incubated with 0.5 mM hydrogen peroxide (H_2_O_2_) for 1 h or 3 h [15]. Control cells were treated with vehicle medium for the same times. All experiments were performed in triplicate.

### 2.3. Histological Analysis

The kidney samples were fixed in 10% formaldehyde, embedded in paraffin, cut into 4 *μ*m sections, and subjected to periodic acid-Schiff (PAS) and Masson Trichrome staining. The pathological changes were observed under a light microscope. Photographs were obtained, and morphology was quantitatively analyzed using the Image-Pro Plus system (Media Cybernetics). The percentage of interstitial collagen deposition was calculated in renal fibrosis [16], and an average of 20 visual fields of each sample was evaluated.

Other kidney sections were used for immunohistochemical analysis. After heat-induced antigen unmasking, sections were incubated with anti-MUTYH antibody or anti-alpha smooth muscle actin antibody for 16 h at 4°C, followed by incubation with secondary antibodies labeled for detection. Other free-floating sections were pretreated as described previously [17] and subjected to 8-oxoG immunodetection in nuclear DNA (nDNA) or mitochondrial DNA (mtDNA). The immunofluorescence images were photographed using a laser scanning confocal microscopy (Zeiss) and the immunohistochemistry slides were recorded using a Nikon light microscope (Nikon Inc). Semiquantitative analysis of MUYTH or 8-oxoG expression was evaluated using the Image-Pro Plus system. The integrated optical density (IOD) in tubules was examined for at least 20 consecutive microscopic fields each section. The percentage of tubules with MUTYH immunoreactivity was also evaluated in a mean (+SE) of 281 (+11) tubules.

### 2.4. Immunofluorescent Analysis* In Vitro*

The cultured HK-2 cells were first washed with PBS and fixed in 4% paraformaldehyde for 20 min at 4°C. Following three washes with PBS (5 min each), cells were permeabilized with 0.5% Triton X-100 for 10 min, blocked in 5% BSA for 30 min, and incubated overnight at 4°C with and anti-MUTYH or anti-8-oxoG antibody in PBS in a dark chamber, followed by secondary antibody incubation. The special pretreatment for 8-oxoG staining was conducted as previously described [17]. Before the addition of a coverslip, the cell slides were incubated with 2 mM DAPI (D1306, Molecular Probes). Fluorescent images were examined by laser scanning confocal microscopy.

### 2.5. TUNEL Analysis

The terminal deoxynucleotidyl transferase-mediated UTP nick end labeling (TUNEL) staining was analyzed with a DNA fragmentation detection kit (11684817910, Roche Corp) according to the manufacturer's instructions. The positive apoptotic cells were detected, and the apoptotic index was calculated as the ratio of positive apoptotic cardiocytes to total number of HK-2 cells. A mean (+SE) of 961 (+147) cells was counted for analysis.

### 2.6. Western Blot Analysis

The kidney tissue and cells were lysed on ice for 30 min using RIPA lysis buffer (P002A, AURAGENE). After boiling with loading buffer, 30 *μ*g of extracted protein was subjected to SDS-PAGE and transferred to PVDF (polyvinyl difluoride) membranes (IPVH00010, Millipore). After blocking the nonspecific binding sites, the primary antibodies rabbit anti-MutY and mouse monoclonal anti-alpha smooth muscle actin were incubated at 4°C overnight, followed by incubation with an HRP-labeled secondary antibody. The bands were detected with an enhanced chemiluminescent reagent (Millipore), and specific ~57 kDa and ~53 kDa bands for MUTYH were observed. Relevant bands were quantified by densitometry using Image J, background corrected and normalized to GAPDH levels.

### 2.7. Statistical Analysis

Data were shown as the mean ± SD and analyzed using SPSS version 13.0. Comparisons were performed using Student's *t*-test for two groups or ANOVA (one-way analysis of variance) for three groups, and a nonparametric test was used when necessary. Two-tailed *P* values less than 0.05 were considered statistically significant.

## 3. Results

### 3.1. Regulation of MUTYH in Two Models* In Vitro*

**Figure 1:**
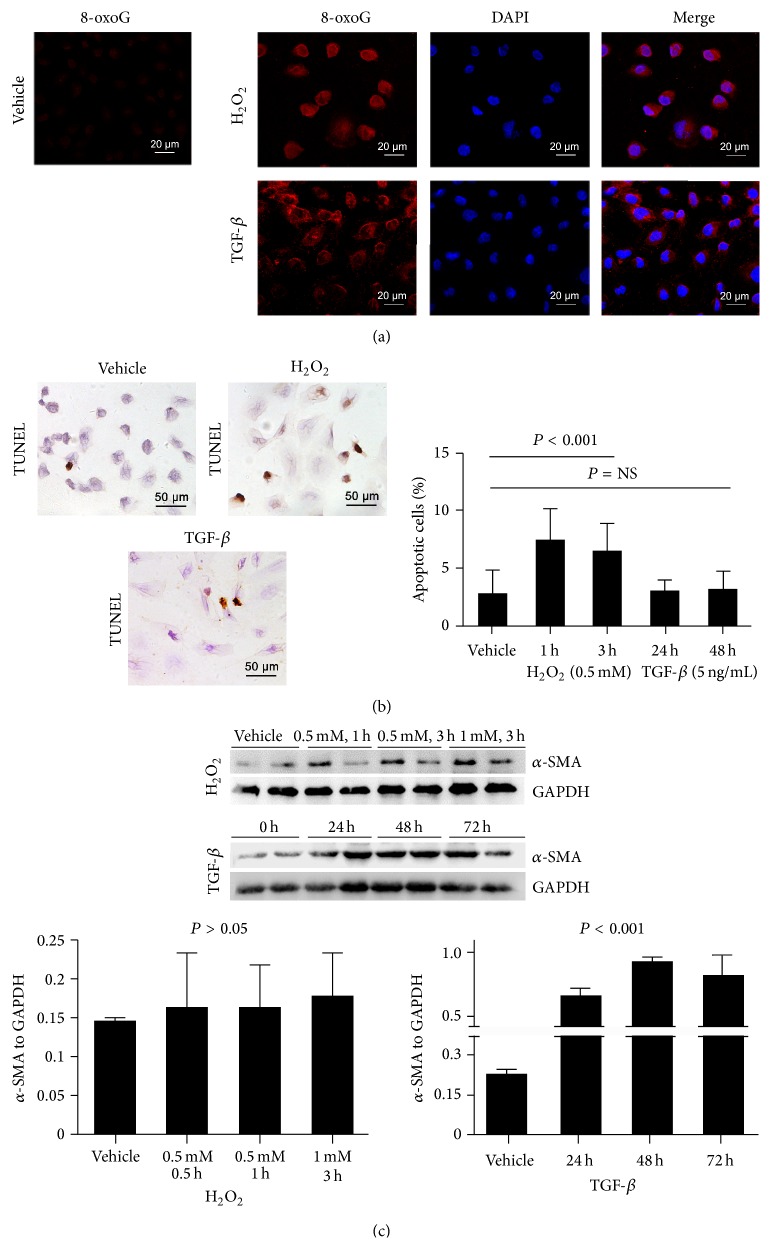
Induced response to H_2_O_2_ and TGF-*β*1 in HK-2. Increased 8-oxoG staining was observed in HK-2 cells induced by H_2_O_2_ and TGF-*β*1 (a). Apoptotic cells with positive TUNEL staining increased after H_2_O_2_ treatment, but not after TGF-*β*1 treatment (b). Increased *α*-SMA expression and EMT in HK-2 cells was induced by TGF-*β*1, but not H_2_O_2_ (c).

To explore the response of MUTYH in renal fibrosis* in vitro*, we analyzed HK-2 cells treated by H_2_O_2_ and TGF-*β*1 [2]. With both H_2_O_2_ and TGF-*β*1 treatments, HK-2 cells showed higher 8-oxoG expression compared with vehicle controls (Figure 1(a)). The immunoreactivity of 8-oxoG was mainly located in HK-2 cell mitochondria and only partly in nuclei.

Different cell responses were observed after H_2_O_2_ or TGF-*β*1 treatment. H_2_O_2_ leads to cell apoptosis, and the percentage of apoptotic cells increased significantly after incubation with 0.5 mM H_2_O_2_ for 1 h and 3 h (Figure 1(b)). TGF-*β*1 only slightly increased cell apoptosis after treatment for 24 h or 48 h (*P* > 0.05). However, *α*-SMA expression increased in HK-2 cells after 24 to 72 h of TGF-*β*1 treatment (Figure 1(c)). The expression of *α*-SMA was unchanged by H_2_O_2_ treatment. These findings suggested that HK-2 cells respond to H_2_O_2_ by cell apoptosis and to TGF-*β*1 by EMT.

**Figure 2:**
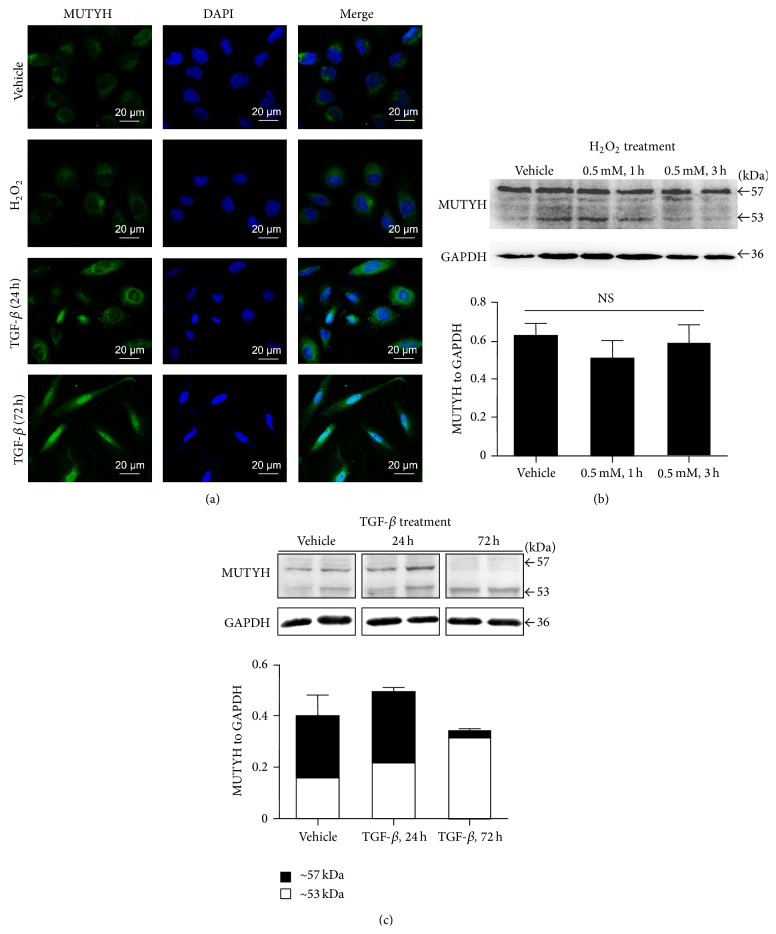
*In vitro* regulation of MUTYH in HK-2 tubular cells treated with H_2_O_2_ and TGF-*β*1. Immunofluorescent images demonstrated the location of MUTYH (in green) compared to DAPI (in blue) staining (a). Immunoblot analysis showed similar expression of MUTYH after H_2_O_2_ treatment (b) and distinct regulation of MUTYH after TGF-*β*1 treatment (c).

H_2_O_2_ and TGF-*β*1 also showed diverse regulation of MUTYH in HK-2 cells (Figure 2). In the vehicle group, HK-2 cells had high mitochondrial MUTYH expression (Figure 2(a), top line) and ~57 kDa bands in Western blotting (Figures 2(b) and 2(c)). After H_2_O_2_ treatment, similar mitochondrial expression was detected in HK-2 cells (Figure 2(a), second line), and semiquantitative analysis demonstrated equal MUTYH expression compared with controls (Figure 2(b)). TGF-*β*1 treatment induced HK-2 cells from a cobblestone-like morphology to a spindle-like morphology, and MUTYH immunoreactivity was located in nuclei (Figure 2(a)). After TGF-*β*1 treatment for 72 h, almost all of the cells demonstrated nuclear MUTYH (Figure 2(a), last line). Western blotting analysis confirmed increased 53 kDa nuclear MUTYH and decreased 57 kDa mitochondrial MUTYH in HK-2 cells after TGF-*β*1 treatment. The concentration of both mitochondrial and nuclear MUTYH in HK-2 cells was also upregulated by TGF-*β*1 in 24 hours (*P* < 0.001, Figure 2(c)).

### 3.2. MUTYH Expression* In Vivo* Kidney of UUO Mice

**Figure 3:**
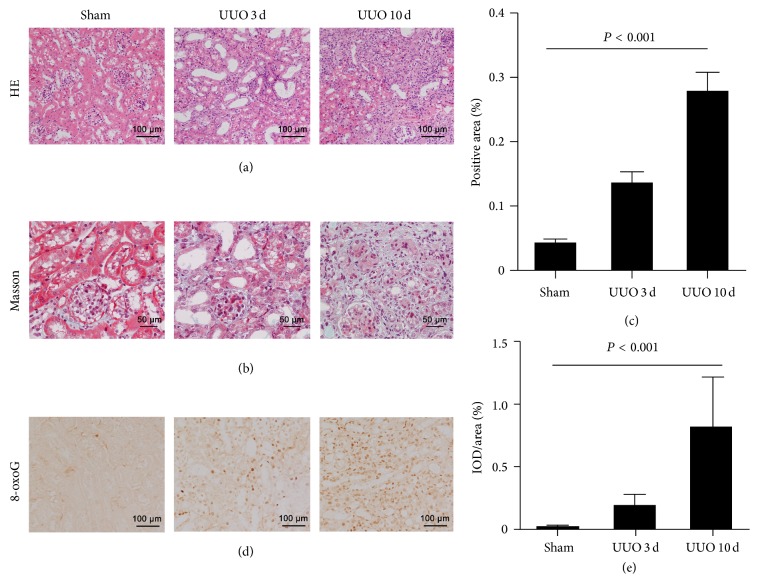
Histological analysis in kidneys from UUO mice. Representative microscopy images of sham-operated kidneys and UUO kidneys for 3 days and 10 days with HE staining (a) and Masson staining (b). Collagen deposition was significantly higher in UUO mice than sham-operated kidneys ((c), *P* < 0.001). The immunoreactivity of 8-oxoG was shown in UUO kidneys (d) and 8-oxoG intensity increased in UUO kidneys compared to sham controls ((e), *P* < 0.001).

The UUO mice at 3 and 10 days after the operation showed proximal tubule dilation, atrophy, and extracellular matrix (ECM) accumulation as a result of collagen deposition. The typical lesions of obstructed kidneys are demonstrated in Figures 3(a) and 3(b). The interstitial collagen deposition was significantly higher in UUO mice than sham-operated kidneys (*P* < 0.001, ANOVA; Figure 3(c)). The 8-oxoG staining was undetectable in sham-operated kidneys, while oxidative DNA lesions were observed in UUO kidneys (Figure 3(d)). The intensity of 8-oxoG staining significantly increased by Day 3 in obstructed kidneys and was even greater at Day 10 (*P* < 0.001, ANOVA; Figure 3(e)) when compared with the shams.

**Figure 4:**
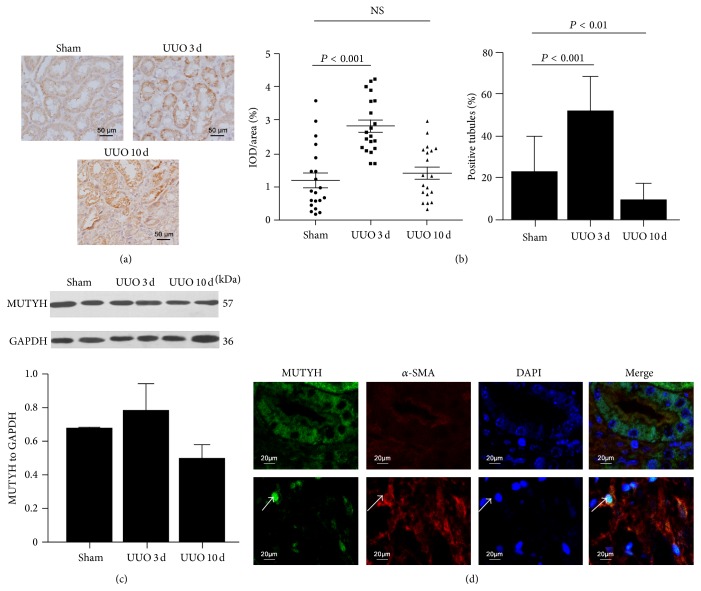
Regulation of MUTYH in renal fibrosis* in vivo*, as shown in UUO kidneys. Immunohistochemical staining of MUTYH showed granular accumulation in tubules of UUO kidneys (a). Both intensity (left panel) and the number of positive tubules (right panel) increased in obstructed kidneys for 3 days and then declined by 10 days ((b), *P* < 0.001). Western blotting showed MUTYH expression in kidneys with dominant mitochondrial bands of ~57 kDa (c). When *α*-SMA (red) was observed ((d), upper panel), MUTYH staining (in green) localized to the cytoplasm, without overlapping DAPI (in blue). Otherwise, when *α*-SMA was highly expressed, a few cells with nuclear MUTYH staining (white arrows) were detected in fibrotic interstitial cells ((d), low panel).

MUTYH immunoreactivity can be observed in sham-operated kidneys, and this increased in some tubules in UUO kidneys but did not in interstitium (Figure 4(a)). The MUTYH density in positive tubules was increased significantly in UUO kidney at Day 3 when compared with sham controls, and percentage of positive tubules was also increased (Figure 4(b)). However, the density of MUTYH and the percentage of positive tubules declined in UUO kidneys between Day 3 and Day 10. Particularly, the great MUTYH staining was observed in the residual obstructed tubules at Day 10, but the number of the residual tubules was decreased compared with sham or Day 3 groups (Figure 4(a), the right panel). It suggested that injured tubules presented increased MUTYH expression, while atrophic ones lost its expression. As injured and atrophic tubules were observed at same time, Western blotting only showed slightly increased MUTYH expression in UUO kidneys at Day 3 and then followed by a decrease at Day 10 (Figure 4(c)).

Moreover, the MUTYH bands were mainly at ~57 kDa (Figure 4(c)), suggesting that the mitochondrial form of MUTYH was mainly expressed in kidney. Correspondingly, the immunofluorescent images showed that MUTYH was mainly located in the cytoplasm of renal tubular epithelial cells (Figure 4(d), upper line), where *α*-SMA was not highly expressed. In fibrotic UUO kidneys, the expression of *α*-SMA increased in the interstitium, and expression of nuclear MUTYH in a few cells was also observed (Figure 4(d), lower line).

### 3.3. MUTYH Immunoreactivity in Human Kidneys

**Figure 5:**
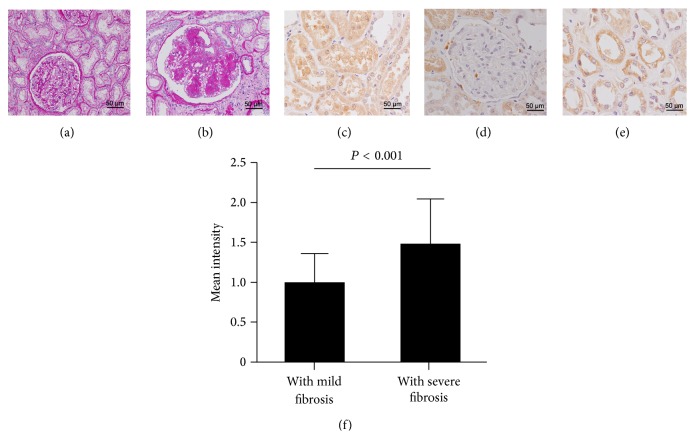
MUTYH immunoreactivity in kidneys from patients with diabetic nephropathy. Representative images from two groups of patients: mild fibrosis (a) and severe fibrosis (b). MUTYH staining was observed in renal tubules (c) but was minimal in glomeruli and interstitium (d). Intense MUTYH staining was visible in tubules in kidneys with severe fibrosis (e) and the IOD (integral optical density) for MUTYH was statistically higher in the severe fibrosis group than in the mild group ((f), *P* < 0.001).

Diabetic nephropathy (DN) is a common kidney disease, presenting with glomerular lesions and tubulointerstitial fibrosis. Kidney samples were divided into two groups: mild renal fibrosis (Figure 5(a)) and severe renal fibrosis (Figure 5(b)). In subjects with mild fibrosis, MUTYH immunoreactivity was observed in renal tubule epithelium (Figure 5(c)), but not in the glomeruli or the interstitium (Figure 5(d)). In kidneys with severe fibrosis, the tubule epithelium showed intense MUTYH immunostaining (Figure 5(e)), while cells in glomeruli and interstitial spaces were rarely observed (not shown). The MUTYH intensity in tubules significantly increased with the degree of renal fibrosis (*P* < 0.001, Figure 5(f)).

## 4. Discussion

In kidneys, accumulating evidence has demonstrated that kidney fibrosis induced by renal diseases, including diabetic nephropathy, is mediated by ROS/oxidative stress [2, 18, 19]. MUTYH is a well-known oxidative DNA repair enzyme which initiates DNA oxidative damage repair by recognizing A:8-oxoG mismatches and removing the mispaired A. This study demonstrated that expression of MUTYH is regulated in renal tubular epithelial cells from renal fibrosis, suggesting that oxidative DNA repair is involved in this process. The mitochondrial isoform of MUTYH was dominant in kidney and acted in a cell-type-specific manner, even within the same tissue type. Additionally, our data demonstrated that upregulation of MUTYH began at an earlier stage after obstruction injury and was sustained with tubular atrophy. This implied that oxidative DNA repair pathway was involved in the renal fibrosis.

MUTYH expression in kidney tubules was shown in patients with DN. Increased MUTYH expression was associated with the severity of renal fibrosis. Detailed information was obtained from UUO models* in vivo*. Murine UUO is a frequently used model of progressive renal injury and fibrosis [20]. In obstructed kidneys, increased MUTYH expression was detected in tubule cells compared with sham-operated kidneys.

We observed an interesting pattern of MUTYH staining with immunohistochemistry analysis. At early stage of lesions, MUTYH expression was increased in tubular cells, while it was decreased with tubular atrophy. The MUTYH expression in interstitial cells seemed not to respond to obstruction injury. It suggested that MUTYH expression in UUO performed a cell-type-specific and stage-dependent response under obstruction injury. Such inconsistence with the same tissues and timely response of expression would result in light change in whole kidney at a specific time, when analyzed by Western blotting analysis. Kim et al. [21] also demonstrated that ROS was harmful, playing a role in proliferation and death in a cell-type-specific way in tubule cells, but not in interstitial cells.

The 8-oxoG lesion is a major marker of oxidative damage and is associated with the progression of renal fibrosis. Antioxidative treatment or blockage of specific pathways will therefore attenuate the fibrotic progress [22]. In the present study, we confirmed that 8-oxoG levels increased in UUO kidneys, related with the severity of renal fibrosis. However, oxidative DNA damage did not parallel MUTYH regulation in the kidney. The 8-oxoG staining increased in both tubule epithelial cells and interstitial cells, whereas MUTYH upregulation was only detected in tubules. Epithelial-mesenchymal transition (EMT) and apoptosis of tubule epithelial cells are two major contributors to renal fibrosis [23]. EMT and apoptosis were induced* in vitro* by TGF-*β*1 and H_2_O_2_, respectively. Both H_2_O_2_ stimulation and TGF-*β*1 stimulation induced increased 8-oxoG in HK-2 cells. However, only TGF-*β*1 treatment leads to MUTYH upregulation. H_2_O_2_, a powerful source of ROS, had no effect on MUTYH expression. These inconsistencies implied that mechanisms in addition to the oxidative DNA damage underlying renal fibrosis were involved in MUTYH regulation.

Moreover, MUTYH has different forms that localize to mitochondria and nuclei, with relative molecular masses of ~57 kDa and ~53 kDa, respectively [10]. During kidney fibrosis, 8-oxoG concentrations increased in both nuclei and mitochondria, and the mitochondrial 8-oxoG was dominant. Similarly, MUYTH was detected in tubule epithelial cell cytoplasm, and increased mitochondrial MUTYH was related to renal fibrosis. Induced expression of nuclear MUTYH was also observed in the fibrotic area labeled by a-SMA staining, but only a few cells were detected* in vivo*. Interestingly, when TGF-*β*1 induced EMT in HK-2 cells, the dominant MUTYH form changed from mitochondrial to nuclear. This finding suggested that both mitochondrial and nuclear forms of MUTYH were involved in renal fibrosis. They seemed to play distinct roles in tubule cell injury, but more evidence is needed.

An accumulation of 8-oxoG and upregulation of MUTYH were observed in tumor tissues [24]. Dysfunction of MUTYH leading to accumulative 8-oxoG and subsequently mutation was previously considered to perform role of disease. Recently, MUTYH triggered single-strand breaks (SSBs) in DNA were suggested to aggravate brain damage and promote neurodegeneration [25]. Suppression of MUTYH may even be protective under oxidative stress. To understand the association of MUTYH polymorphisms with renal fibrosis and ESRD [9], the present study demonstrated MUTYH upregulation in kidneys, which might be involved in EMT in renal fibrosis. Additionally, the oxidative DNA damage might not be the only trigger for MUTYH regulation. Although the results in the present study could not demonstrate the role of MUTYH on renal fibrosis, the significance of this study is by the first time to illustrate the association between MUTYH regulation and renal obstruction. The present study is fundamental for further studies, and our ongoing studies showed that MUTYH deficiency protects mice from renal fibrosis (data not shown). The underlying mechanism concerning SSBs and EMT is being investigated.

Taken together, our results demonstrated the regulation of MUTYH in tubule cells in renal fibrosis. Renal fibrosis caused a cell-type-specific and time-dependent response of oxidative DNA repairs, even within the same tissues. There are several reports on MUTYH in cancer research, but few on renal diseases. To our knowledge, this is the first report to describe MUTYH in the kidneys, and it provides a new insight into renal fibrosis. More studies will focus on the regulatory role of MUTYH in the kidney and provide target validation for therapeutic strategies.
